# One health insights to prevent the next HxNy viral outbreak: learning from the epidemiology of H7N9

**DOI:** 10.1186/s12879-019-3752-6

**Published:** 2019-02-11

**Authors:** Zhe Zheng, Yi Lu, Kirsty R. Short, Jiahai Lu

**Affiliations:** 1 School of Public Health, Sun Yat-sen University, Zhongshan 2nd Road, Guangzhou, 510080 Guangdong China; 2 Department of Environmental Health Sciences, School of Public Health, University at Albany, State University of New York, 1 University Place, Rensselaer, NY 12144 USA; 3 Key Laboratory of Tropical Disease Control, Sun Yat-sen University, Zhongshan 2nd Road, Guangzhou, Guangdong China; 4 One Health Center of Excellence for Research &Training, Zhongshan 2nd Road, Guangzhou, Guangdong China; 5 School of Chemistry and Molecular Biosciences, The University of Queensland, QLD, St Lucia, 4072 Australia; 6 Australian Infectious Diseases Research Centre, The University of Queensland, QLD, St Lucia, 4072 Australia

**Keywords:** H7N9, One health, Zoonosis, Influenza virus, Disease management

## Abstract

**Background:**

With an increased incidence of viral zoonoses, there is an impetus to strengthen collaborations between public health, agricultural and environmental departments. This interdisciplinary cooperation, also known as the ‘One Health’ approach, has received significant support from various stakeholders. However, current efforts and policies still fall short of those needed for an effective One Health approach towards disease control and prevention. The avian-origin H7N9 influenza A virus outbreak in China serves as an ideal case study to emphasise this point.

**Discussion:**

Here, we present the features and epidemiology of human infections with H7N9 influenza virus. At the early stages of the H7N9 epidemic, there was limited virus surveillance and limited prevention measures implemented in live poultry markets. As a result, zoonotic infections with H7N9 influenza viruses continued to enlarge in both numbers and geographic distribution. It was only after the number of human infections with H7N9 influenza virus spiked in the 5th wave of the epidemic that inter-departmental alliances were formed. This resulted in the rapid control of the number of human infections. We therefore further discuss the barriers that prevented the implementation of an effective One Health approach in China and what this means for other emerging, zoonotic viral diseases.

**Summary:**

Effective implementation of evidence-based disease management approaches in China will result in substantial health and economic gains. The continual threat of avian influenza, as well as other emerging zoonotic viral infections, emphasizes the need to remove the barriers that prevent the effective implementation of One Health policies in disease management.

## Background

**Fig. 1:**
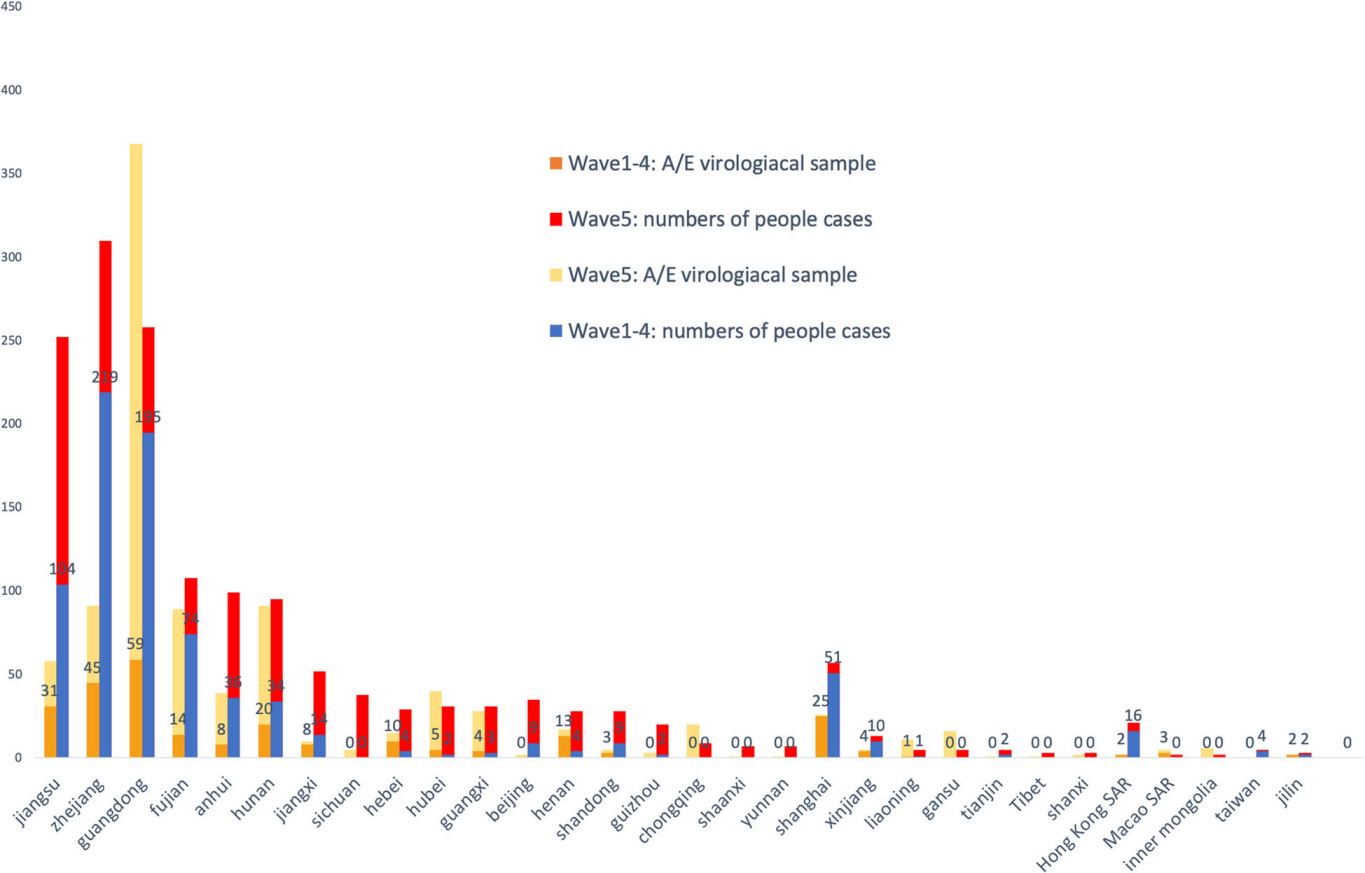
Number of reported human cases and positive virological samples from birds or the environment, by province and origin as of March 2018. Data include both high and low pathogenic H7N9 viruses. The yellow charts indicate the number of positive virological samples from birds or the environment of the total of the first four waves. The orange charts indicate that of the five waves. The shallow green charts stand for the number of the total reported human cases of the first four waves and the deep green ones stand for that of the five waves. The five waves almost count the sum up of the first four waves both in human and animal. Poultry data comes from FAO [53]. Human data comes from WHO [1]

Human infections with avian influenza virus H7N9 were first identified in March 2013 in south-eastern China. The virus then spread rapidly to the inner provinces of China. As of March 2nd 2018, 1567 laboratory-confirmed cases of human infected with avian influenza A(H7N9) viruses have been reported to the World Health Organization (WHO), including at least 615 deaths (a fatality rate of 39%) [1]. The absence of overt clinical signs in poultry [2], and the lack of effective intervention strategies by the agricultural departments, led to a large number of viral infections in poultry and six discreet ‘waves’ of human infections [3]. The first H7N9 epidemic wave started in March and ended in June 2013. In subsequent waves (2013–2018), the H7N9 influenza virus infection season typically started in October and lasted until the following June. From October 2016 to June 2017, the number of human H7N9 influenza virus infections increased to 759 cases (almost the same as the sum of the previous four waves) and spread across 23 provinces and 6 municipalities [4]. During this wave, a highly pathogenic avian influenza (HPAI) virus A (H7N9) was identified in birds from the live poultry markets in Guangdong [5]. Subsequently, thousands of chickens died as a result of HPAI virus H7N9 infection [6, 7]. These events prompted the Chinese Ministry of Agriculture (MoA) to issue an emergency notice, asking to strengthen national H7N9 prevention and controls [8, 9]. This cross-disciplinary effort from numerous government department led to a rapid decline in the number of human H7N9 influenza virus infections and only 3 cases have been reported since July 2017 [1]. The comparison between former four epidemic waves and wave fifth in both human and poultry are shown in Fig. 1.

Cross-species transmission and the evolution of the H7N9 virus from a low pathogenic to high pathogenic phenotype emphasizes the importance of One Health – which is based upon the premise that there is an intimate relationship between human, animal and environmental health. Only when we maintain this broader perspective can we hope to prevent the next endemic/pandemic with an HxNy influenza virus. In this article, we will use the outbreak of H7N9 influenza viruses in humans as an example of the benefits of implementing a rapid and comprehensive One Health approach for emerging zoonotic infections.

## Discussion

### Influenza H7N9 virology

Influenza A virus H7N9 is a negative sense RNA virus with two key surface glycoproteins, the haemagglutinin (HA) and the neuraminidase (NA). It is capable of undergoing both antigenic drift (driven by point mutations) and antigenic shift (as a result of co-infection and genome reassortment). The NA of H7N9 influenza viruses isolated from human cases most closely resembles that found in migrating birds from South Korea [10]. In contrast, the HA of human H7N9 influenza viruses can be traced back to the HA of an H7N3 influenza virus from domestic ducks [11]. Typically, the HA of avian influenza viruses shows a strong preference to bind to α-2,3 linked sialic acids whilst the HA of human influenza viruses typically binds to α-2,6 linked sialic acid [12]. Since the upper respiratory tract of humans is rich in α-2,6 receptors, human influenza viruses are well adapted for efficient human-to-human transmission [13]. However, via the introduction of point mutations in viral HA (Q226L/I, G186 V), the avian H7N9 influenza virus increased its avidity to human receptors [13]. Thus, it breached the species barrier and was able to spread from poultry to humans [14–16]. By retaining its ability to bind to α-2,3 linked sialic acid, the virus predominately replicated in the lower respiratory tract, limiting the efficacy of person-to-person spread [17]. This dual receptor specificity allowed the virus to continue to circulate in poultry, including chickens and quails [18, 19].

In the first four waves of the H7N9 outbreak, the cleavage site of the viral HA possessed only a single amino acid R (arginine), indicating a low pathogenic phenotype in poultry [11, 20]. However, in viruses isolated from live poultry markets (LPM) in Guangdong province during the fifth wave, researchers found four amino acids (RKRT) inserted into the HA cleavage site (4 of 69 strains). This insertion was associated with the conversion of the virus from a low to high pathogenic phenotype in poultry [21]. Moreover, an increased number of virus strains with a 588 V mutation in the PB2 were detected [22]. The 588 V mutation has previously been associated with enhanced viral pathogenesis in mammalian species [22, 23]. Indeed, there were at least 23 different genotypes of H7N9 influenza viruses detected by the end of December 2017. Among those, seven genotypes were genetically distinct, whilst 16 were found to have evolved from the 2013 H7N9 influenza viruses [21]. Although the HPAI virus H7N9 was first isolated from LPMs in Guangdong, it is believed that the HPAI virus H7N9 originated from the Yangtze river delta and spread to the southeast coast via live poultry transactions [24]. The HPAI virus H7N9 then evolved into multiple genotypes via reassortment with H9N2 influenza viruses and local low pathogenic H7N9 viruses [24]. As of July 2017, the HPAI H7N9 influenza virus has spread to more than 12 different provinces in China [24].

### Epidemiology of human infections with H7N9 influenza viruses

**Fig. 2:**
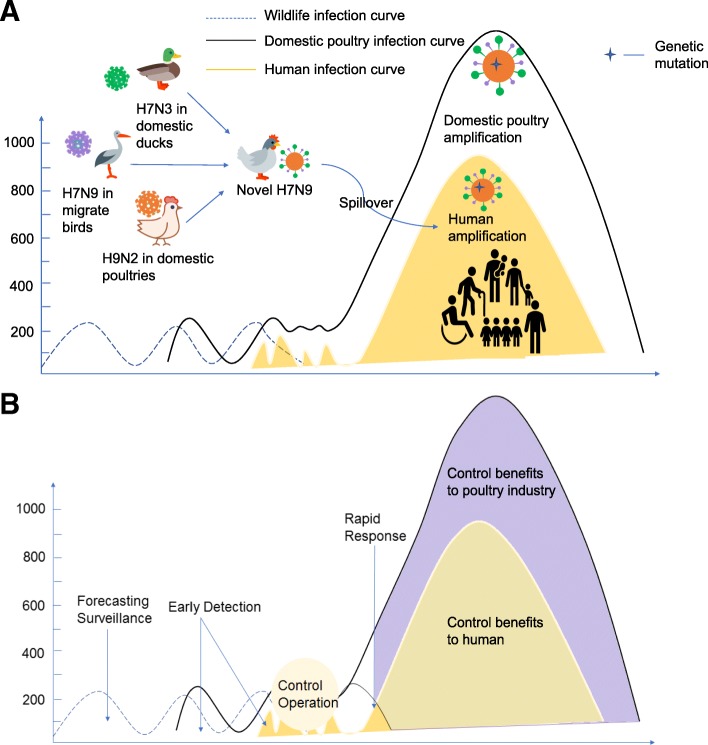
H7N9 virus origin and its development. **a** illustrates the current H7N9 epidemic. Sequencing analyses revealed that the human infections with H7N9 virus came from three avian origins, with six internal genes from avian influenza A (H9N2) viruses in domestic poultries, the hemagglutinin (HA) gene from AI H7N3 in domestic ducks and the neuraminidase (NA) gene mutate from AI H7N9 in migrating birds reservoir [11]. After they recombined and mutated into the novel LPAI H7N9 virus that can infect poultry with little to no disease in poultry, it came into the human-animal interface and then gained the power to cross the species barrier. Causing four epidemic waves in human and poultry, the H7N9 transmit wider in the chicken reservoir, adding the opportunities to mutate and recombine. With genetic mutation, H7N9 virus amplify among both poultry and human population in the five wave. **b** illustrates the control benefits we would gain if implement One Health policy

Since February 2013, six waves of human H7N9 influenza virus infections have occurred in China. The total number of human infections has varied dramatically between waves, ranging from 134 cases in the first wave to 781 cases in the fifth wave. Only 3 cases have been reported since October 2017 to present day (i.e during the sixth wave). Like other influenza virus strains, the H7N9 influenza virus showed increased circulation amongst both poultry and humans in winter and spring months, in particular from December to April. In China, more than half of the reported cases have been concentrated in Zhejiang (310), Guangdong (258) and Jiangsu (251) provinces [4]. Three distinct stages were noted during the H7N9 influenza virus epidemic. The first stage was characterized by sporadic human infections with the virus (waves one to four of the epidemic). However, since October 2017, once the virus became established in poultry populations across China, the number of human H7N9 influenza virus cases surged. This second stage was associated with a reduction in the mean patient age, suggesting that a larger proportion of the population was at risk [25–27]. Fortunately, following a joint effort by animal and public health authorities, both human and poultry infections were controlled, and the number of human cases remains limited (Fig. 2).

Among all human H7N9 influenza virus infections recorded to date, almost 50% of cases have occurred in individuals over the age of 60. It is also striking to note that the majority of infections have occurred in males. Retired individuals are likely to have a higher risk of virus exposure, as the elderly frequently visit LPMs. Indeed, approximately 80% of patients exposed to poultry did so at a LPM, whilst occupational exposure only accounted for approximately 10% of patients [3, 28, 29]. The role of LPMs in the zoonotic spread of H7N9 influenza viruses is further supported by the fact that positive viral samples are most commonly isolated from chickens sold in LPMs [30]. Moreover, sequence analysis of H7N9 viruses isolated from LPMs suggests that poultry are the most likely source of human infections [11]. This is consistent with experimental studies showing that H7N9 virus titers are higher in quails and chickens (when compared to other poultry), and only these two species can efficiently transmit the virus through direct contact [31]. In contrast, pigs are unable to sufficiently transmit the H7N9 influenza virus after experimental infection [18]. Poultry appeared to have played a particularly significant role in the H7N9 epidemic outbreak as they surpassed human-source predictors to be the dominant factor in disease outbreaks [23].

### H7N9 prevention and control measures in China

During the first four waves of the epidemic, the H7N9 virus was classified as a LPAI virus and caused no overt clinical symptoms in poultry. This impeded attempts to develop accurate viral surveillance [32]. Moreover, in light of the associated economic losses, some agricultural authorities and members of the poultry industry contested the conclusion that poultry were the source of human infections [33]. These individuals emphasized that there was insufficient evidence to prove that the poultry were the original source of the virus [34]. It was further argued that the H7N9 influenza virus should neither be named as avian influenza nor should the government report every human infection to the general public [34, 35]. This dissent represented a significant obstacle for information transparency and inter-departmental collaboration.

**Fig. 3:**
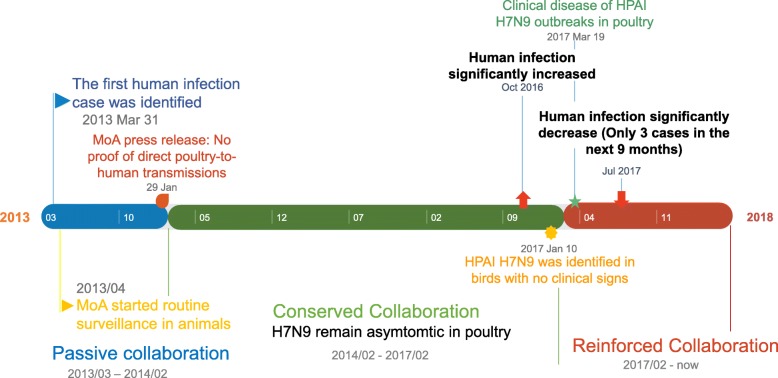
Government measures time-line. Agricultural departments and public health departments turn from reserved collaboration to reinforced collaboration

When human infections with the H7N9 influenza virus were first identified, control strategies focused on patient isolation and health education [36]. As more information became available about the transmission of the H7N9 virus, disease control departments focused on strengthening disease prevention and surveillance amongst poultry industry workers, enhancing the sanitation of LPMs and improving hygiene during poultry slaughter and transactions [37, 38]. Simultaneously, the MoA established active surveillance in animals and the environment at multiple sites whilst the Chinese Centers of Disease Control (CDC) isolated and identified the virus from patients and tested aerosol samples from LPMs [39, 40]. The control strategies used at LPMs during this time included flushing once per day, sterilizing once per week and closing once per month so that a thorough disinfection process could be carried out [41]. LPMs were also closed when human and/or poultry cases were reported. After the HPAI virus H7N9 was identified and caused significant poultry morbidity and mortality, animal and public health authorities organized various conferences to bring together veterinary, epidemiology and virology experts. These conferences helped establish strong inter-departmental collaborations to prevent and control the spread of H7N9 influenza viruses [42]. This approach proved to be highly successful and only 3 cases of human infection with H7N9 influenza virus were reported in the last wave (Fig. 3).

### The one health approach

Today, due to the the increasingly blurry human-animal interface, zoonotic infections are increasing at an unprecedented rate [43]. This interdependence between human health, animal health, and environmental health gave rise to the concept of “One Health”, which means solving the growing problem of infectious disease via cooperation between experts in different disciplines [44]. As the world’s biggest agricultural country, with a large human-animal interface, China experiences a large number of emerging infectious zoonotic diseases, [45, 46]. This feature makes China the ideal setting for the implementation of a One Health policy and to effectively address these health challenges.

### Current impediments

**Fig. 4:**
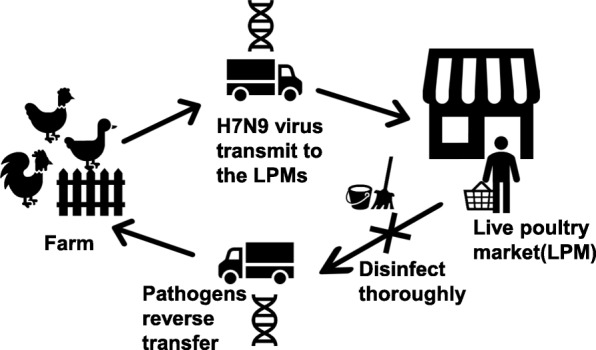
H7N9 virus transmission routes. H7N9 virus maintain in poultry reservoirs in farms and backyard. During transportation, virus transmits among the large group of live poultries through direct and/or indirect contact. Arriving at LPMs, immunological naive population exposes to infected poultries and/or H7N9 virus aerosol. Risk groups get affected. Contaminated cages and other staffs may transfer H7N9 virus reversely to farms and backyards and form an infection loop. Frequent clean and disinfect after each workday could cut down the loop

China plays a vital role in the international poultry trade [47]. However, 30% of the poultry raised in China are raised in backyard conditions without any biosecurity [48]. This widespread use of backyard farms makes it challenging for the MoA to conduct routine avian diseases surveillance. Furthermore, the unique trade model used in China, “Company + peasant household”, creates opportunities for H7N9 influenza viruses to transmit from poultry farms to wholesale markets, where different genotypes avian influenza viruses can reassort. There is also a risk of reverse virus transmission from LPMs to farms [15, 31]. However, because the rate of positive serology samples is much higher in LPMs than farms, agricultural departments may neglect this dual transmission pattern, resulting in continued infections [30] (Fig. 4).

Another impediment to controlling the H7N9 influenza virus outbreak is the protection of sectional interests. Eliminating avian influenza in poultry represents a significant economic loss for the poultry industry. Initially, farmers and local authorities were reluctant to report avian influenza outbreaks, in particular, the asymptomatic LPAI virus H7N9 cases. Some farmers and small companies even transported infected poultry away from their premises once a viral infection had been identified, further facilitating the spread of the virus [49]. The difficulties associated with monitoring/preventing short distance, live poultry transportations potentially contributed to the repeated waves of H7N9 influenza virus outbreaks in China.

To a certain extent, closing LPMs after the start of an avian influenza virus epidemic can reduce the risk of human zoonotic infections [50]. However, such an approach should only be viewed as a temporary measure as it does not eliminate the source of the infection. Moreover, LPMs form an integral part of some people’s livelihood and it would be unrealistic to permanently close LPMs. Moreover, even if the government banned live poultry transactions in markets (and only permitted the sale of frozen chickens) many people would simply sell and buy live poultry on an individual basis. This would make it even harder to establish a consolidated disease control management program and it would significantly hamper any efforts to investigate poultry exposure history [42].

### Using one health to prevent the next HxNy influenza virus outbreak

The prevention and control of H7N9 influenza virus infections represents an important case-study in One Health. During the first few waves of the epidemic, there was no consolidated, multidisciplinary response and the outbreak continued unabated. However, the number of human H7N9 virus infections were successfully controlled when all the relevant stakeholders were able to collaborate and form a united response. New zoonotic influenza A viruses like the H7N9 virus will continue to emerge from the human-animal interface. Therefore, we propose the following four ways to strengthen the cooperation between agricultural departments and public health departments in the context of zoonotic disease outbreaks.

1. The early deployment of multi-sectoral joint prevention and control mechanisms is essential in the early detection and control of epidemics. The agricultural sector and the disease control department need to conduct instant information interchanges and adopt joint monitoring and disposal measures. Establishing and improving animal transportation and supervision mechanisms, such as animal border inspection and quarantine stations in provinces and major cities, will also help to avoid the ongoing spread of the disease [51].
2. Strengthened biosecurity (i.e. segregation, cleaning and disinfection) in both farms and LPMs. This could include establishing biosecurity isolation zones to prevent the spread of new avian viruses via the contact of poultry and wild birds. The government should further focus on standardizing the poultry industry and enhancing farming support. This could include promoting industrial transformation and improving cold chain transportation systems. After early detection of live poultry infections, culling, disinfection, isolation and other virus purification measures must be carried out immediately [52].
3. Continued active monitoring of the wild bird reservoirs and intensified virus tracing. Diagnostic teams in the veterinary system should use high-throughput viral sequencing to continually monitor and analyze the diversity of DNA/RNA viruses at the human-animal interface. These technologies will extend the scope of virus surveillance, identify potential emerging infectious diseases and improve early warning capabilities. Establishing such a rapid virus monitoring and reporting system will facilitate more efficient source control of the disease.
4. Develop new influenza virus vaccines and antiviral drugs and prioritize their distribution. This approach should include prioritizing vaccination of exposed animal workers and domestic animals. This requires us to move from the current passive monitoring system in hospitals to the ‘front line’. Stopping and monitoring the infection of those at occupational risk of exposure is essential in protecting the general population.

## Summary

Since 2018, through the concerted efforts of the disease control department and the agricultural sector, the number of human cases of H7N9 avian influenza virus infection has fallen sharply. This is a clear example of the importance of a One Health strategy in combating zoonosis. To better control H7N9 influenza virus and prevent the emergence of novel influenza virus strains, we advocate establishing coordination and cooperation among local governments, veterinarians, disease control and market management departments. This will ensure that we are able to effectively meet the constant challenge of emerging infectious diseases.

## Abbreviations

CDC: Centers of disease control
HA: Haemagglutinin
HPAI: High pathogenic avian influenza
LPAI: Low pathogenic avian influenza
LPM: Live poultry market
MoA: The Ministry of Agriculture China
NA: Neuraminidase
SA: Sialic acids
WHO: World Health Organization
